# Photocatalytic Degradation of Organic Pollutants over MFe_2_O_4_ (M = Co, Ni, Cu, Zn) Nanoparticles at Neutral pH

**DOI:** 10.1038/s41598-020-61930-2

**Published:** 2020-03-18

**Authors:** Nishesh Kumar Gupta, Yasaman Ghaffari, Suho Kim, Jiyeol Bae, Kwang Soo Kim, Md Saifuddin

**Affiliations:** 1 University of Science and Technology (UST), Daejeon, Republic of Korea; 2 Department of Land, Water, and Environment Research, Korea Institute of Civil Engineering and Building Technology (KICT), Goyang, Republic of Korea

**Keywords:** Catalysis, Environmental chemistry, Inorganic chemistry

## Abstract

In this study, we report a surfactant-mediated synthesis of ferrites (MFe_2_O_4_: M = Co, Ni, Cu, Zn) using the co-precipitation-oxidation method. The band gap calculated from UV-Visible diffuse reflectance spectra were found in the range of 1.11–1.81 eV. These ferrite nanocatalysts were studied for the photocatalytic degradation of multiple organic dyes in a 32 W UV-C/H_2_O_2_ system. All the four ferrites showed an excellent dye degradation rate in the range of 2.065–2.417 min^−1^ at neutral pH. In the optimized condition, NiF was found to degrade 89%, 92%, 93%, and 78% of methylene blue, methyl orange, bromo green, and methyl red, respectively within 1 min of UV-irradiation. A 40% TOC removal was recorded after 5 min of degradation reaction, which increased to 60% after 50 min. Mechanism elucidated by scavenger studies and fluorescence spectroscopy revealed that ^•^OH and holes were the primary reactive radicals responsible for the degradation process. Ferrite photocatalysts showed an insignificant performance loss in seven consecutive cycles. The photocatalyst was found efficient in the presence of a high concentration of salts. Thus, it was concluded that these photocatalysts are highly suitable for the remediation of dye-contaminated wastewater.

## Introduction

The ever-increasing population, coupled with rapid urbanization and industrialization, have deteriorated the quality of life. Excessive contamination of water bodies could be a severe threat to both human beings and other life forms. Organic dyes are considered as one of the major pollutants discharged into the environment by textile, printing, food, and leather industries^1^. Though a large segment of these synthetic dyes is non-toxic or less toxic, their presence in water increases the oxygen demand, which in turn affects aquatic animals^2^. Among the dyes consumed in industries, up to 70% belong to azo dye family (those with azo-functional group “‒N = N‒”). Azo dyes have genotoxic, mutagenic, and carcinogenic effects on living beings^3^. Some of the azo dyes are known to be carcinogenic in the non-cleaved state, and for many of the azo dyes, their cleaved products such as benzidine are known to induce tumors^4^. Conventional biological, chemical, and physical methods like adsorption^5,6^, chemical precipitation^7^, and microbial degradation^8^ have been established for the remediation of dye-contaminated wastewater. The practical application of these processes suffers due to high operational cost, sludge production, or formation of secondary pollutants. For a complete or partial degradation of organic dyes (into non-toxic byproducts), degradation by adopting the photocatalytic process is one of the viable options.

In recent years, advanced oxidation processes based on the generation of highly reactive hydroxyl radicals have gained momentum for the degradation of toxic organic pollutants^9^. Though the Fenton process is easier to operate, it suffers badly due to a slow regeneration of Fe^2+^ ions^10^. Thus, the ultraviolet radiation or electrochemical method is generally coupled with the Fenton process for the regeneration of Fe^2+^ ions^10,11^. Unfortunately, the homogeneous photo-Fenton process has two significant drawbacks, i.e., a narrow operational pH range and formation of sludge, which increase the overall cost of the process^12^. These drawbacks are motivating researchers to develop cost-effective UV-light responsive heterogeneous photocatalysts.

Among numerous metal-oxide based photocatalysts developed, researchers are fixated on spinel ferrites due to their narrow band gap, magnetic property, and high stability^13^. Moreover, the magnetic and optical properties of Fe_3_O_4_ (simplest ferrite) could be easily tuned by replacing Fe^2+^ ions with other divalent cations (Mn, Co, Ni, Cu, Zn, Ca, and Mg)^14^. Many researchers have exploited ferrite photocatalysts for the photocatalytic degradation of organic pollutants. Cai *et al*., 2016 developed ZnFe_2_O_4_ via a reduction-oxidation method, which showed decolorization of Orange II dye in visible light/catalyst/H_2_O_2_ system^15^. Sharma *et al*., 2014 synthesized MFe_2_O_4_ (M = Co, Ni, Cu, Zn) by sol-gel method and studied the degradation of methylene blue at pH 2.5 in visible light/catalyst/H_2_O_2_ system^16^. Dhiman *et al*., 2016 reported visible light-assisted photocatalytic degradation of safranine-O and remazol brilliant yellow at pH 2.5 onto morphologically different NiFe_2_O_4_ synthesized by hydrothermal route^17^. Though these studies showed remarkable visible-light-driven photocatalytic degradation of organic dyes, significant issues with these research works were low pH requirement, slow degradation kinetics, and high energy consumption.

Considering these drawbacks as challenges, we focused on the development of novel ferrite photocatalysts, which could be used for dye degradation at neutral pH with low energy consumption. We successfully developed an economical and robust surfactant-mediated co-precipitation method for the fabrication of spinel ferrites (MFe_2_O_4_; M = Co, Ni, Cu, Zn). The prepared photocatalysts were characterized by various microscopic and spectroscopic techniques for understanding the structural, functional, and optical properties. These ferrite photocatalysts were found highly efficient in degrading multiple organic dyes at neutral pH in a 32 W UV-C/H_2_O_2_ system. The kinetic rates observed in the present study were the highest ever reported under these sets of experimental conditions. Furthermore, the dye degradation mechanism was deduced based on the scavenger studies and fluorescence spectroscopy.

## Results and discussions

### Characterization of ferrite photocatalysts

The morphology of ferrite photocatalysts was characterized by scanning electron microscopy (SEM), and results have been shown in Supplementary Fig. 1. SEM micrographs of all the four photocatalysts, i.e., CoF, NiF, CuF, and ZnF showed a coral-like morphology. EDS analysis of CoF photocatalyst has been shown in Supplementary Fig. 2. From the EDS spectrum in Supplementary Fig. 2(a), Fe, Co, and O were observed at their corresponding keV values. The peaks of Pt observed in the EDS spectrum were due to the coating at the time of SEM analysis for better visibility of the surface morphology. The 2D elemental mappings showed a uniform distribution of constituent elements in CoF, thus confirming the homogeneity of the photocatalyst (Supplementary Fig. 2(b)).

**Figure 1:**
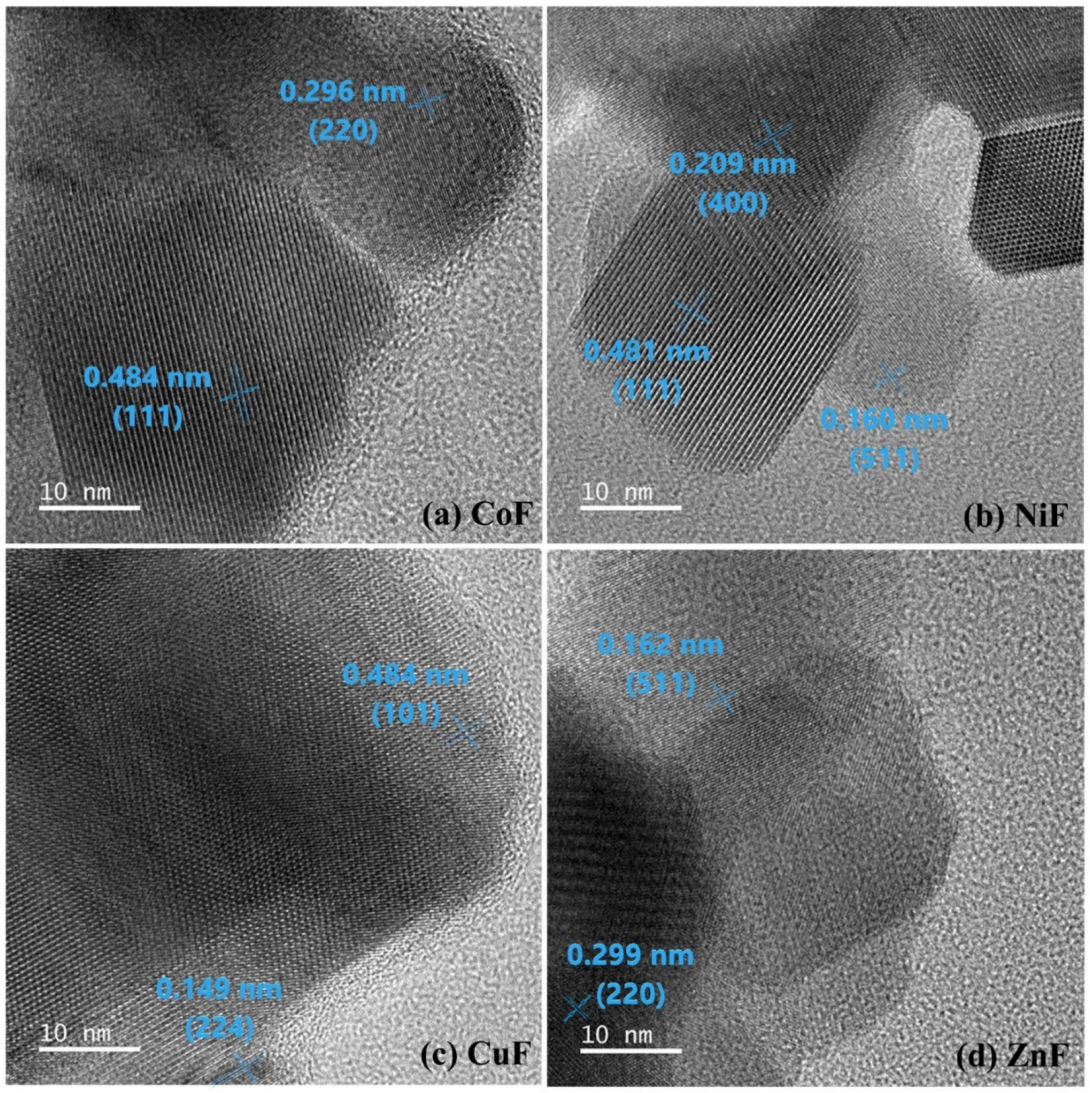
HRTEM images of (**a**) CoF (**b**) NiF (**c**) CuF (d) ZnF photocatalysts.

The morphology of ferrites was further investigated by high-resolution transmission electron microscopy (HRTEM). Single crystals were observed for all the four ferrites (Supplementary Fig. 3). The crystallite planes were assigned by measuring the fringe width values on the HRTEM image and then correlating it with the interplanar spacing (*d*) values from the XRD pattern. The fringe width of 0.48 nm corresponding to the (111) plane was observed for both CoF and NiF ferrites. The same fringe width was also observed for CuF, but it was assigned to the (101) plane of the tetragonal crystal system. For ZnF, the two fringes width, 0.299 nm (220), and 0.162 nm (511) were distinctly visible (Fig. 1).

Porosity properties and specific surface area of ferrite nanoparticles were analyzed by N_2_ adsorption-desorption isotherm (Supplementary Fig. 4). All the four ferrites showed a similar hysteresis loop at a high relative pressure fit for type IV of adsorption isotherm corresponding to the mesoporous materials^18^. The hysteresis loop at a high relative pressure (*P*/*P*_0_) in the range of 0.5–1.0 showed that the mesopores are irregular in the photocatalyst which was further confirmed by the Barrett, Joyner, and Halenda pore size distribution (inset images in Supplementary Fig. 4). The three photocatalysts, i.e., CoF, NiF, and CuF, showed a broader pore size in the range of 3–250 nm. In contrast, for ZnF, pore size in the range of 3–60 nm with a narrow peak centered at ~35 nm showed non-uniform sizes and shapes of the nanopores from the agglomeration of ferrite nanoparticles. The method for calculating total surface area (*S*_tot_) and BET surface area (*S*_BET_) of ferrites nanoparticles has been given in Supplementary Section 1. The *S*_BET_ for ferrites in the range of 13–23 m^2^ g^−1^ were found lower than those reported in the literature where ferrites were synthesized by sol-gel method^16^. Various parameters like *S*_tot_, *S*_BET_, pore volume (*V*_p_), and pore diameter (*D*_p_) have been tabulated in Supplementary Table 1.

**Figure 2:**
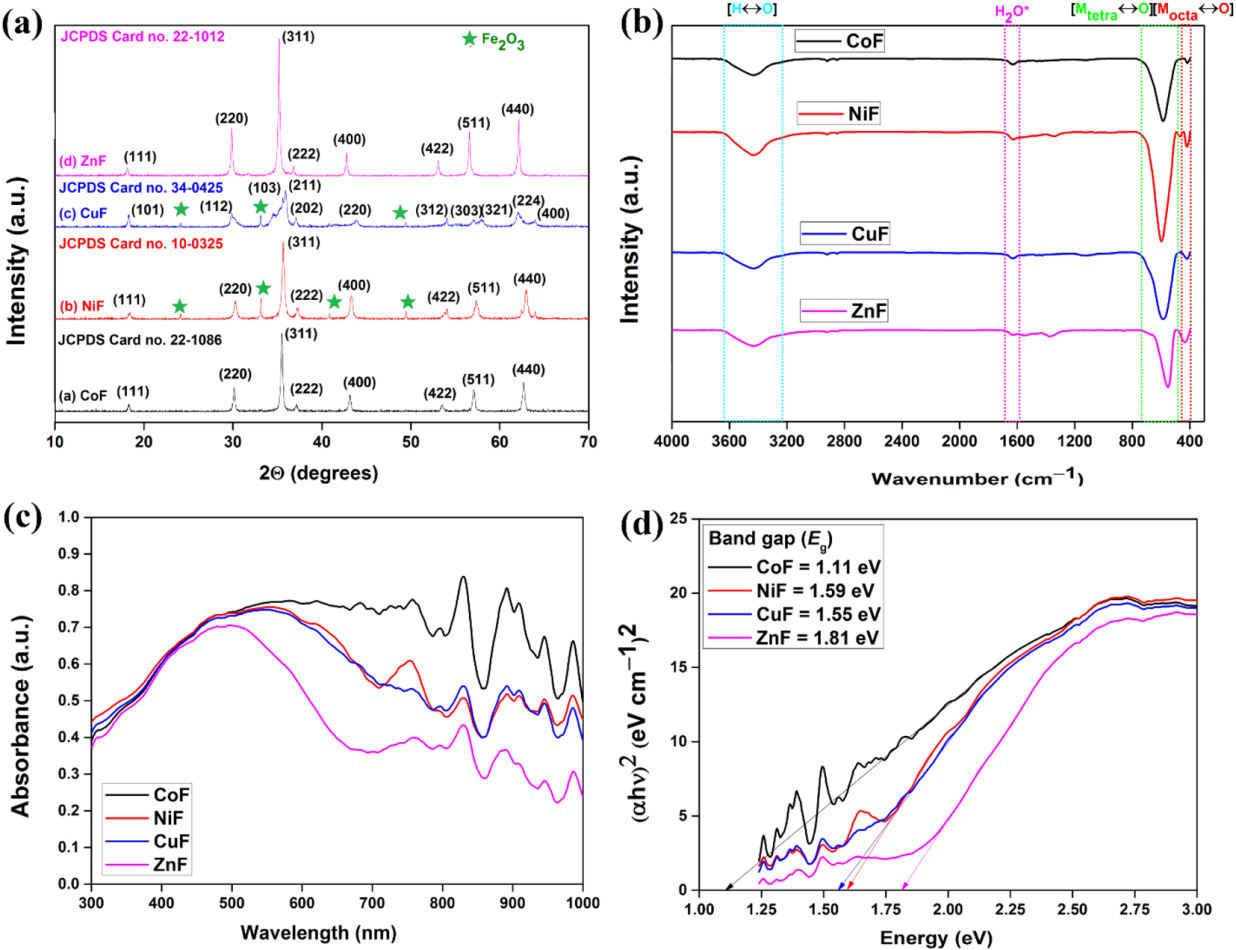
(**a**) XRD spectra; (**b**) FTIR spectra; (**c**) UV-Vis DRS spectra; (**d**) band gap energies of ferrites photocatalysts.

Figure 2(a) showed the X-ray diffraction (XRD) pattern of CoF, NiF, CuF, and ZnF. The diffraction peaks of CuF nanoparticles at 18.3°, 29.9°, 35.3°, 35.9°, 37.1°, 43.9°, 54.1°, 57.1°, 58.0°, 62.1°, and 64.0° were assigned to (*hkl*) (101), (112), (103), (211), (202), (220), (312), (303), (321), (224), and (400) planes of tetragonal CuFe_2_O_4_ (JCPDS card no. 34–0425)^19^. The lattice parameters, *a* (5.83 Å) and *c* (8.61 Å), matched well with the literature^20^. Besides, the diffraction peaks appeared for CoF, NiF, and ZnF matched with the JCPDS card no. 22–1086, 10–0325, and 22–1012, respectively. The lattice parameter for these cubic spinel structures, *a* = 8.38 Å (CoF), 8.34 Å (NiF), and 8.45 Å (ZnF), was found consistent with the earlier reported values^16^. In NiF and CuF photocatalysts, Fe_2_O_3_ was confirmed as an impurity that was absent in CoF and ZnF. The crystallite size of a ferrite nanoparticle was calculated using the Debye-Scherrer equation: *D* = 0.9*λ*/(*β* Cos*θ*), where *D* is the crystallite size, *λ* is the wavelength of Cu K*α* radiation, *β* is the full width at half maximum of the diffraction peak, and *θ* is the Bragg angle^21^. The crystallite size for CoF, NiF, CuF, and ZnF was found to be 34 nm, 27 nm, 16 nm, and 36 nm, respectively (Supplementary Table 2).

Figure 2(b) showed the Fourier-transform infrared (FTIR) spectra of CoF, NiF, CuF, and ZnF photocatalysts. Two distinct bands for metal-oxygen vibrations were observed in the range of 750–400 cm^−1^ for all the four ferrite photocatalysts. The high-intensity band in the range of 750–500 cm^−1^ (*v*_1_) was attributed to the intrinsic stretching vibrations of the tetrahedral site-occupied metal-oxygen bond [M^2+^_tetra_ ↔ O]. The low-intensity band 450–400 cm^−1^ (*v*_2_) was due to the octahedral Fe^3+^-oxygen stretching vibration^21^. The broad band centered at 3427 cm^−1^ and a low-intensity band centered at 1630 cm^−1^ were assigned to the stretching and bending mode of O–H bonds of physically adsorbed water molecules.

Ultraviolet visible diffuse reflectance spectroscopy (UV-Vis DRS) analysis was performed to evaluate the optical properties of ferrite photocatalysts, and results have been shown in Fig. 2(c). All four ferrites showed a strong absorption band in the entire UV-Visible region. The optical direct band gap was calculated using the following equation^22^:

1 $${({\alpha }{h}{\upsilon })}^{2}=B(hu-{E}_{g})$$

where, *α*, *υ*, and *B* are absorption coefficient, light frequency, and the proportionality constant, respectively. The (*αhυ*)^2^ versus *hυ* plots for ferrite photocatalysts have been shown in Fig. 2(d). The band gap calculated for CoF, NiF, CuF, and ZnF, i.e., 1.11 eV, 1.59 eV, 1.55 eV, and 1.81 eV, respectively, were found significantly lower than those reported in the literature^16^.

**Figure 3:**
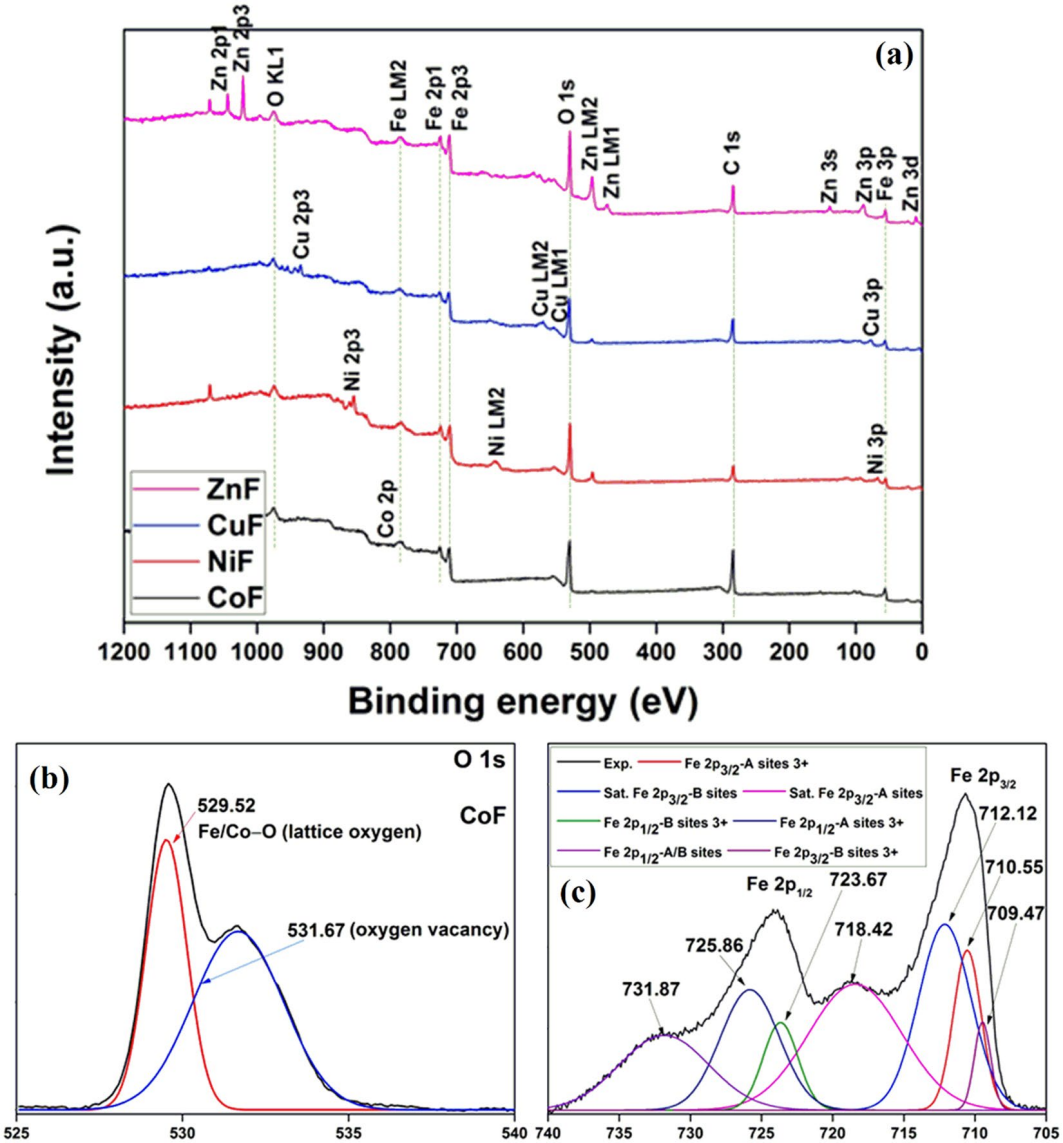
(**a**) XPS full scan; (**b**) HRXPS O 1s spectrum (CoF); (**c**) HRXPS Fe 2p spectrum (CoF).

X-ray photoelectron spectroscopy (XPS) analysis was carried out to determine the elemental composition and oxidation states of ferrite photocatalysts. The XPS full scan spectra of ferrites (Fig. 3(a)) confirmed the existence of predominant constituent elements. The high-resolution XPS (HRXPS) spectra of individual elements have been deconvoluted using Fityk software. In the deconvoluted HRXPS O 1 s spectrum (Fig. 3(b)), two peaks at 529.52 eV and 531.67 eV were assigned to the metal-oxygen bonds (lattice oxygen) and oxygen defect sites, respectively^23^. The proportion of oxygen defect sites was found significantly higher for CoF photocatalyst (image not shown here). In the HRXPS Fe 2p spectrum (Fig. 3(c)), two spin-orbit doublets Fe 2p_3/2_ (709.47 eV for B-sites and 710.55 eV for A-sites) and Fe 2p_1/2_ (723.67 eV for B-sites and 725.86 eV for A-sites) were observed for Fe^3+^ ions in the ferrite photocatalyst. The peaks at 718.42 eV, 723.67 eV, and 731.87 eV were satellite peaks^24^. The peak position of Fe 2p_3/2_ B-sites and Fe 2p_1/2_ B-sites coincided with the peak positions of Fe^2+^ species^25^, indicating that Fe^2+^/Fe^3+^ ions could occupy the octahedral sites. The HRXPS Co 2p spectrum (Supplementary Fig. 6(a)) showed two peaks at 782.06 eV and 787.53 eV, which were due to the existence of Co^2+^ ions^26^. For the HRXPS Ni 2p spectrum (Supplementary Fig. 6(b)), two spin-orbit doublets corresponding to the characteristic peaks of Ni^2+^ (853.61 eV) and Ni^3+^ (855.23 eV) and two shakeup satellites (860.44 eV and 878.19 eV) were observed showing a peculiar existence of Ni^3+^ in the NiF photocatalyst^27^. Supplementary Figure 6(c) showed the binding energies of Cu 2p_3/2_ (933.90 eV) and Cu 2p_1/2_ (953.76) with two satellites confirming the presence of Cu^2+^ ions in CuF photocatalyst^28^. Supplementary Figure 6(d) showed two peaks at 1020.37 eV and 1043.50 eV for Zn^2+^ corresponding to Zn 2p_3/2_ and Zn 2p_1/2_, respectively for ZnF photocatalyst^29^.

The electron spin resonance (ESR) spectrum of ZnF photocatalyst has been shown in Supplementary Fig. 7. In ZnF, two systems contribute toward ESR signals: one is high spin Fe^3+^ (*d*^5^) coordinated with oxygen, and the other is singly ionized oxygen vacancy (V_O_^+^). The doubly ionized (V_O_^+2^) and non-ionized (V_O_^0^) oxygen vacancies are ESR silent^30^. The Zn^2+^ ions with filled *d*^10^ orbital do not contribute to the ESR signal. In the present case, a signal was recorded at *g* = 2.001, which was assigned to V_O_^+^. The signal at *g* = 2.232 for Fe^3+^ ion in the octahedral site of spinel ferrite was not observed. The signal was masked by the signal at *g* = 2.001, which could be due to the effect of calcination of ferrite photocatalyst at 700 °C^31^.

### Photocatalytic activity of ferrites

**Figure 4:**
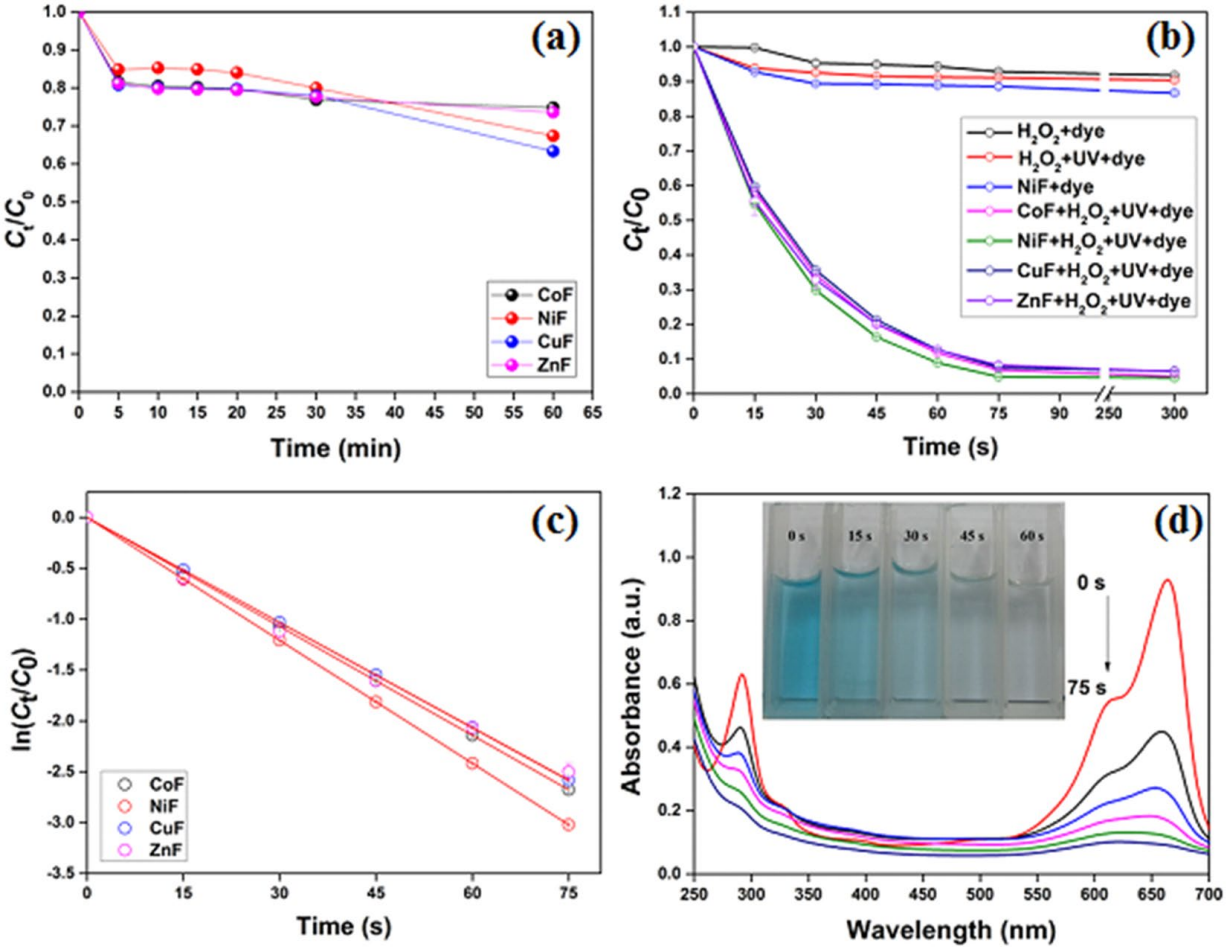
Degradation of MB dye over ferrite photocatalysts. (**a**) UV irradiation; (**b**) photocatalytic degradation in H_2_O_2_/UV system (**c**) plot of ln(*C*_t_/*C*_0_) versus time (*t*); (**d**) intensity change with irradiation time (ZnF). Conditions: [MB] = 10 mg L^−1^, [photocatalyst] = 0.5 g L^−1^, [H_2_O_2_] = 5 mmol L^−1^.

The photocatalytic activity of all the four ferrites, i.e., CoF, NiF, CuF, and ZnF, was found unsatisfactory in the absence of H_2_O_2_ and only 30–35% of dye was found to degrade after 60 min of UV irradiation (Fig. 4(a)). Unfortunately, the photogenerated electron-hole pairs were less effective for the process due to fast recombination. In the absence of photocatalyst, less than 8% of dye degradation was observed with H_2_O_2_. In the presence of UV and H_2_O_2_, nearly 10% decolorization was recorded without any photocatalyst. In the dark condition, nearly 14% of MB dye was adsorbed onto NiF after 5 min of agitation. Thus, it was evident that the contribution of adsorption in photocatalytic degradation of dye would be even lesser due to faster degradation kinetics. The H_2_O_2_-assisted photodegradation of organic dye over ferrite photocatalysts showed rapid decolorization at neutral pH. As shown in Fig. 4(b), more than 90% of MB dye was found to degrade within 75 s of irradiation over ferrite photocatalysts. The high-intensity peak at 664 nm is due to conjugation between two dimethylamine substituted aromatic rings through S and N. In contrast, the low-intensity peak in the ultraviolet region (~292 nm) appears due to the aromatic rings. From Fig. 4(d), it was evident that along with the fast decolorization of MB dye-containing solution (due to complete breakdown of the chromophore), the intensity of 292 nm peak also decreased with time without forming any new band in the UV or visible region. Thus, it was concluded that complete structural degradation of MB dye was possible in UV-C/H_2_O_2_/ferrite system. The photocatalytic degradation follows the pseudo-first-order kinetic model (Eq. 3) which could be expressed mathematically as^32^:

2 $$\mathrm{ln}\left(\frac{{C}_{t}}{{C}_{0}}\right)=-\,{k}_{app}t$$

3 $${t}_{1/2}=\frac{0.693}{{k}_{app}}$$

where *C*_0_ and *C*_t_ are the MB concentration at time *t* = 0 and time ‘*t*’, respectively, and *t*_1/2_ is the half-life of the dye degradation. The slope of the *ln*(*C*_t_/*C*_0_) versus *t* plot is the apparent rate constant for the reaction (*k*_app_). From Fig. 4(c), the calculated *k*_app_ value was 2.141 min^−1^, 2.417 min^−1^, 2.065 min^−1^, and 2.069 min^−1^, for CoF, NiF, CuF, and ZnF, respectively. The kinetic rate of degradation for ferrite photocatalysts in this study was found significantly higher than those reported in the literature^32,33^. The parameters evaluated from the kinetic model, along with the linear regression value, have been displayed in Supplementary Table 3.

**Figure 5:**
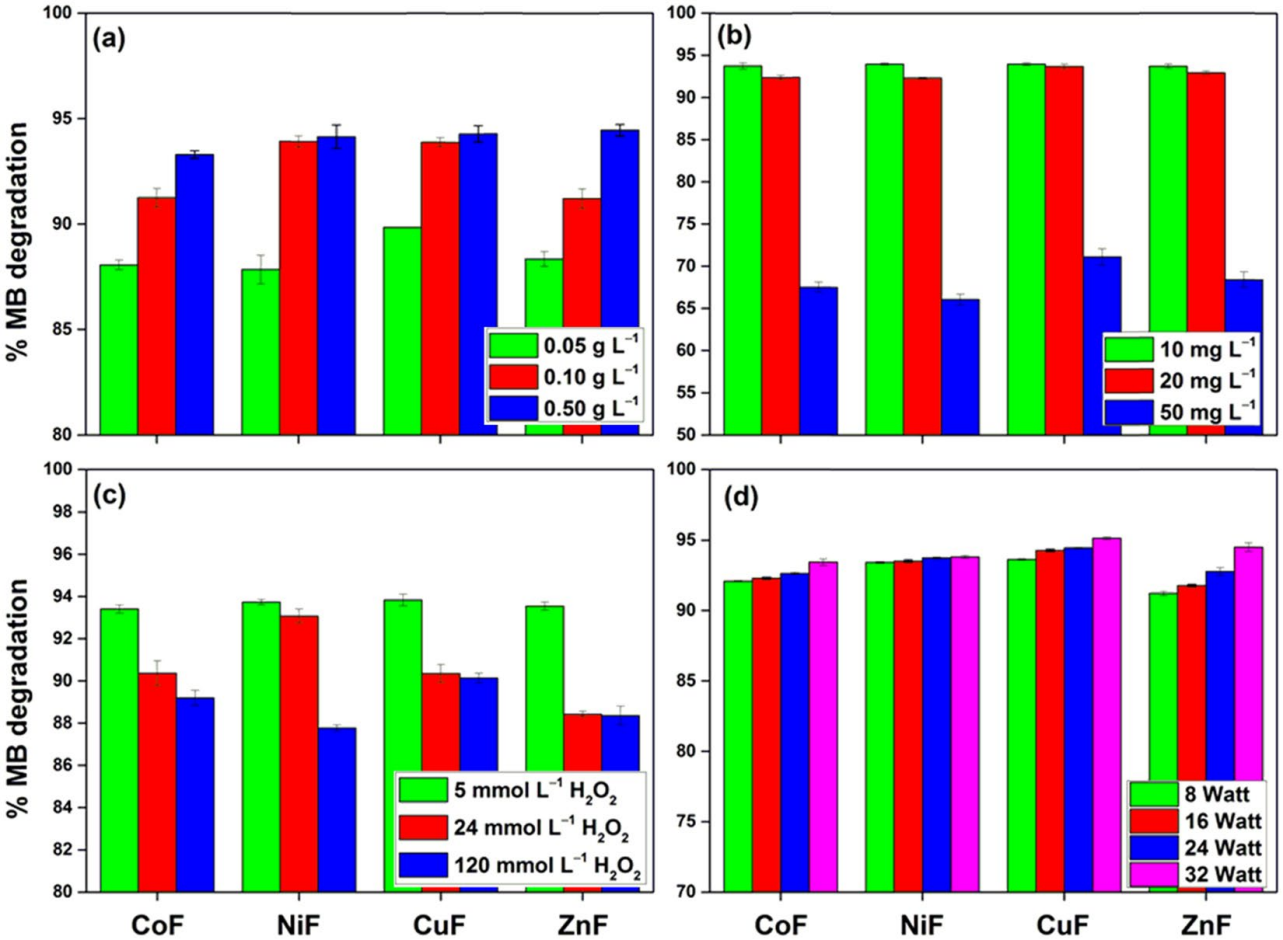
Effect of (**a**) Photocatalyst dosage; (**b**) Dye concentration; (**c**) H_2_O_2_ concentration; (**d**) UV-C power on the degradation of MB dye onto ferrite photocatalysts. Conditions: [MB] = 10 mg L^−1^, [photocatalyst] = 0.5 g L^−1^, [H_2_O_2_] = 5 mmol L^−1^, UV-C power = 16 W, time = 5 min (changed accordingly).

Figure 5(a) shows the effect of photocatalyst loading on the MB degradation under the defined experimental conditions for MFe_2_O_4_ (M = Co, Ni, Cu, Zn). Increasing the photocatalyst dosage from 0.05 g L^−1^ to 0.50 g L^−1^ yielded a slight increase in the %dye degradation performance. The increased photocatalyst dosage favored the degradation process by providing more active sites for the generation of active radicals. Moreover, a small dosage of 0.05 g L^−1^ was found enough for ~90% of MB degradation. Since the maximum dye degradation efficiency was recorded for 0.5 g L^−1^ dosage, subsequent experiments were performed with 0.5 g L^−1^ of the photocatalyst.

In the defined experimental conditions, high degradation efficiencies (~92–94%) were recorded for 10–20 mg L^−1^ solution (Fig. 5(b)). Whereas, it dropped to ~65–70% for a 50 mg L^−1^ MB solution. The primary reason for the result obtained was the limited production of active radicals, which were insufficient to degrade the higher concentration of MB dye^34^. Also, highly concentrated MB dye solution could decrease the penetration of photons in the solution phase and thus hampered with the photogeneration of electron-hole pairs^35^.

It was observed that with the increase in the H_2_O_2_ concentration, the MB degradation efficiency decreased by ~3–5% for all the photocatalysts (Fig. 5(c)). In general, an increased H_2_O_2_ concentration favors the process by providing ^•^OH radicals. Nevertheless, the increased H_2_O_2_ concentration disfavored the photocatalytic process due to the generation of hydroperoxyl radicals (HOO^•^), which exhibits lower oxidation capabilities and did not contribute to the degradation process^16^ (Eqs. 4 and 5). Moreover, radical-radical reactions could have competed with the radical-dye reactions and led to a decreased degradation performance^15^ (Eq. 6). Thus, H_2_O_2_ concentration of 5 mmol L^−1^ was found suitable for the dye degradation process.

4 $$H{O}^{\cdot }+{H}_{2}{O}_{2}\to {H}_{2}O+HO{O}^{\cdot }$$

5 $$HO{O}^{\cdot }+H{O}^{.}\to {H}_{2}O+{O}_{2}$$

6 $$H{O}^{\cdot }+H{O}^{\cdot }\to {H}_{2}{O}_{2}$$

For an energy-efficient process, the power required for the dye degradation was optimized by taking the number of 8 W UV lamps into consideration. As shown in Fig. 5(d), more than 90% of the dye was found to degrade under one lamp of 8 W. But with the increase in the number of lamps from two to four, the photocatalytic performance reached up to 95%. An increased UV power led to the adsorption of more photons by the photocatalysts, which in turn accelerated the formation of electron-hole pairs. Considering the power-to-performance ratio, 16 W, i.e., two 8 W lamps, were taken as the illumination source for subsequent studies.

**Figure 6:**
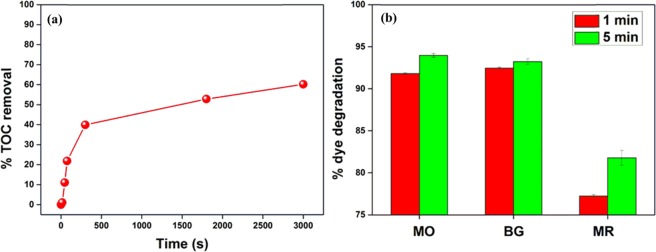
(**a**) The TOC removal efficiency of MB dye using NiF photocatalyst. (**b**) Degradation of MO, BG, and MR dyes. Conditions: [Dye] = 10 mg L^−1^, [H_2_O_2_] = 5 mmol L^−1^, [NiF] = 0.5 g L^−1^, UV-C power = 16 W.

TOC analysis was done to evaluate the mineralization efficiency of a ferrite photocatalyst. The %TOC removal performance of NiF photocatalyst has been shown in Fig. 6(a). In the first 15 s of the degradation process, only 1.1% mineralization of organic species was observed. It increased to 40.0% and 60.2% after 5 min and 50 min, respectively. Thus, it was confirmed that MB dye could be effectively mineralized into residual organic molecules in UV/H_2_O_2_/NiF system.

The optimized parameters were used for the photocatalytic degradation of methyl orange (MO), bromo green (BG), and methyl red (MR) over NiF photocatalyst. Similar experiments were performed, and the photocatalytic performances have been shown in Fig. 6(b). Within 1 min of the irradiation time, nearly 91.8% of MO, 92.5% of BG, and 77.2% of MR were degraded by NiF photocatalyst, which increased to 94.0% (MO), 93.3% (BG), and 81.8% (MR) after 5 min. Thus, these ferrite photocatalysts in the UV-C/H_2_O_2_ system exhibited remarkable properties for rapid degradation of toxic organic dyes at neutral pH.

### Photocatalytic degradation mechanism

**Figure 7:**
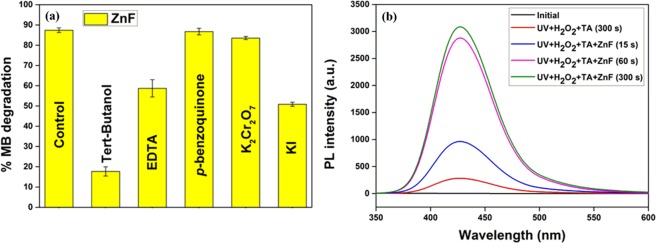
(**a**) Photocatalytic degradation of MB dye over ZnF photocatalyst in the presence of scavengers; (**b**) Fluorescence spectra of 2-hydroxy terephthalic acid (TAOH) (λ_ex_ ~315 nm) in the presence of ZnF.

Different scavenging experiments were performed to identify the main active species responsible for the photocatalytic degradation of organic dyes onto ferrite photocatalysts. EDTA, K_2_Cr_2_O_7_, and *p*-benzoquinone were used as a scavenger for the hole (*h*^+^), electron (*e*^−^), and superoxide anion radical (^•^O_2_^−^), respectively. Tert-butanol was chosen as a hydroxyl radical scavenger for all ^•^OH generated in the reaction process, whereas KI was used as a surface-bound ^•^OH (^•^OH _surf_) scavenger at the catalytic surface^36,37^. As evident from Fig. 7(a), the degradation performance decreased in the order: tert-butanol > KI > EDTA > K_2_Cr_2_O_7_ > *p*-benzoquinone > no scavenger. Since the presence of tert-butanol severely affected the photocatalytic process, it was conclusive that the generated ^•^OH radicals were the primary active species for the degradation process. Additionally, the presence of KI lowered the degradation performance by ~40% within 1 min, suggesting that surface-bound (^•^OH_surf_) released into the solution and took part in the dye degradation process. When ferrite photocatalyst was irradiated under UV lamps, electron/hole (*e*^‒^/*h*^+^) pairs were generated on the surface (Eq. 7). The primary pathway for the generation of ^•^OH radicals was the Fenton reaction, where H_2_O_2_ was activated by regenerated Fe^2+^ (Eq. 8). The regeneration of was possible due to the reaction of Fe^3+^ ions with photogenerated *e*^−^ (*E*^0^ (Fe^3+^/Fe^2+^) = +0.77 V) in the conduction band (Eq. 9). The dye degradation performance decreased from ~89% to ~54% in the presence of EDTA, suggesting the involvement of *h*^+^ (photogenerated in the valence band of ZnF) directly in the degradation of MB dye (Eq. 12). The dye degradation process was also affected by the scavenging behavior of K_2_Cr_2_O_7_ and *p*-benzoquinone. The photogenerated *e*^−^ in the conduction band of ZnF reacted with the adsorbed O_2_ molecules to yield ^•^O_2_^‒^ (Eq. 10), which either reacted directly with the MB dye (Eq. 13) or further combined with H^+^ to produce ^•^OH radicals. From the study, it was conclusive that ^•^OH (Eq. 11) and *h*^+^ served as the major active species, whereas electron was minor active species.

7 $$ZnF+hv\to {e}_{CB}^{-}+{h}_{VB}^{+}$$

8 $${F}{{e}}^{2+}+{H}_{2}{O}_{2}\to F{e}^{3+}+H{O}^{\cdot }+H{O}^{-}$$

9 $$F{e}^{3+}+{e}_{CB}^{-}\to F{e}^{2+}$$

10 $${O}_{2}+{e}_{CB}^{-}\to {O}_{2}^{-\cdot }$$

11 $$H{O}^{\cdot }+Dye\to C{O}_{2}+{H}_{2}O+degradation\,products$$

12 $${h}_{VB}^{+}+Dye\to {[Dy{e}^{+}]}^{\ast }\to degradation\,products$$

13 $${O}_{2}^{-\cdot }+dye\to degradation\,products$$

**Figure 8:**
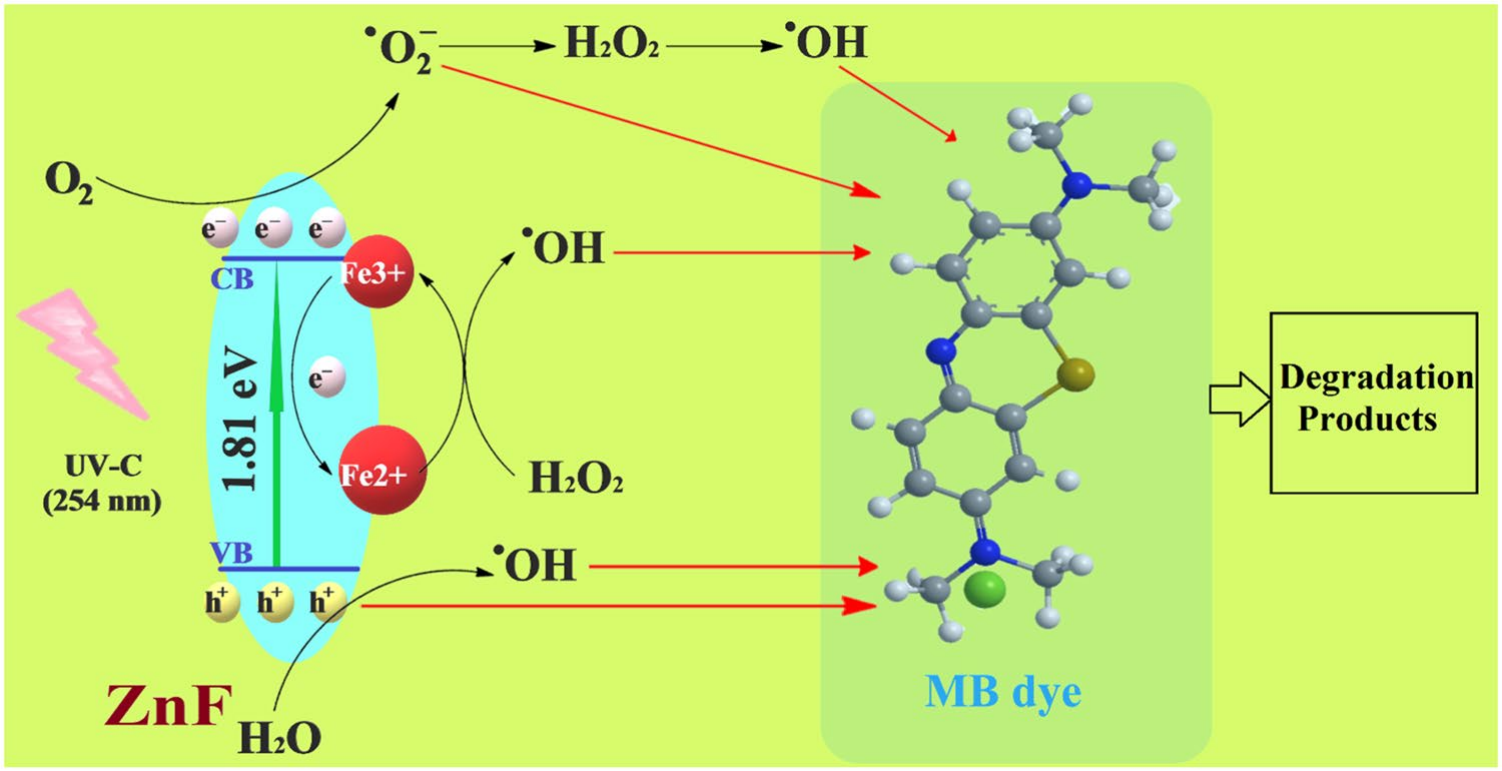
Schematic illustration of photocatalytic degradation process of MB dye onto ZnF photocatalyst.

The formation of ^•^OH radicals on the surface of ZnF photocatalyst in the UV/H_2_O_2_ system was further probed by fluorescence spectroscopy using terephthalic acid (TA) as a probe for ^•^OH radicals. The non-fluorescent TA molecule reacts with an ^•^OH radical to form fluorescent TAOH. The fluorescent intensity of TAOH is directly proportional to the formation of ^•^OH radicals in the photocatalytic reaction and is expected to increase with the irradiation time^38^. When the solution was excited at λ_ex_ ~315 nm, the fluorescence emission was observed at λ_ex_ ~425 nm for TAOH (Fig. 7(b)). Some ^•^OH radicals were generated by UV irradiation of H_2_O_2_ via homolytic cleavage, which translated as a low fluorescence emission in the absence of ZnF photocatalyst. In the presence of ZnF, the fluorescence intensity increased with the irradiation time and reached the maximum after 5 min. Thus, it was conclusive that the formation of ^•^OH radicals primarily drove the photodegradation process. A schematic illustration of the photocatalytic degradation mechanism of MB dye onto ZnF has been shown in Fig. 8.

### Effect of anions and reusability

**Figure 9:**
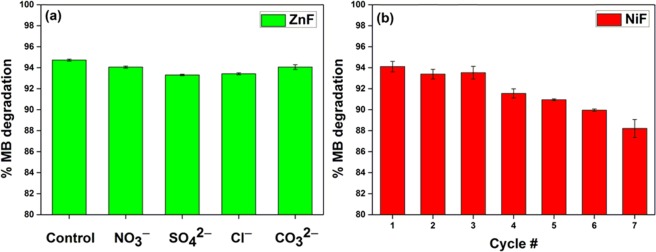
(**a**) Effect of anions on the degradation of MB dye onto ZnF photocatalyst. Conditions: [MB] = 10 mg L^−1^, [anion] = 100 mg L^−1^, [H_2_O_2_] = 5 mmol L^−1^, [photocatalyst] = 0.5 g L^−1^, time = 5 min; (**b**) Reusability of NiF photocatalyst for seven successive cycles for the degradation of MB.

The degradation of MB dye in a photocatalytic process is strongly influenced by the presence of anions such as SO_4_^2−^, NO_3_^−^, Cl^−^, CO_3_^2−^, etc.^39^. The effect of inorganic anions was evaluated by subjecting MB solution with 100 mg L^−1^ concentration of anions to the photodegradation process. The %MB degradation efficiency was found to be 94.7%, 94.1%, 93.3%, 93.4%, and 94.1% for no anion, NO_3_^−^, SO_4_^2−^, Cl^−^, and CO_3_^2−^, respectively (Fig. 9(a)) showing that anions have an insignificant effect on the dye degradation efficiency of ZnF photocatalyst. The reusability of photocatalysts is required for their practical application on an industrial scale. Here also, the photocatalytic performance of NiF photocatalyst was evaluated for seven cycles by regenerating photocatalyst only by washing and drying at 100 °C. In Fig. 9(b), NiF photocatalyst inevitably showed a higher degree of stability and photocatalytic activity towards the degradation of MB dye for seven consecutive runs. Nearly 6.0% loss in photodegradation efficiency was observed after the seventh cycle making these photocatalysts highly suitable for even more cycles.

## Conclusions

In this study, we have demonstrated excellent photocatalytic activity of MFe_2_O_4_ nanoparticles synthesized by the CTAB-mediated co-precipitation-oxidation method. The XRD and TEM analysis confirmed cubic symmetry for CoF, NiF, and ZnF with crystallite size in the range of 27–36 nm. The CuF nanoparticles were in the tetragonal symmetry with 16 nm as the crystallite size. All the ferrites absorbed effectively in the UV-Vis-NIR region. The ferrites were used for the photocatalytic degradation of organic dyes in neutral pH conditions. In optimized experimental conditions, NiF showed an extraordinary rate of 2.417 min^‒1^ for MB dye degradation, which decreased to 2.065 min^‒1^ for ZnF. The NiF photocatalyst showed 89%, 92%, 93%, and 78% decolorization of MB, MO, BG, and MR, respectively, in the same experimental conditions. A 40% TOC removal was observed in the first 5 min of the degradation process, which further increased to 60% after 50 min. The dye degradation mechanism in the UV/H_2_O_2_/ferrite system was primarily driven by the formation of ^•^OH radicals and holes, which was confirmed by scavenger studies and fluorescence spectroscopy. Apart from being highly reusable, the photocatalyst showed excellent performance in the presence of a ten-fold concentration of salts. From this study, it was concluded that these photocatalysts have the potential to be used for the affordable treatment of dye-contaminated wastewater without modifying the pH under low power UV-C irradiation.

## Methods

All the chemicals were of analytical grade and used without any further purification, whose details are available in Supplementary Section 2. MFe_2_O_4_ NPs were synthesized by the surfactant-mediated co-precipitation method^40^. For the synthesis of CoFe_2_O_4_, 4.76 g of CoCl_2_•6H_2_O and 16.16 g of Fe(NO_3_)_3_•9H_2_O were dissolved in 500 mL of distilled water at 60 °C with constant stirring for 10 min. To it, 1.0 g cetyl trimethylammonium bromide was added and vigorously stirred at 60 °C for 30 min. To this solution, 1.0 mol L^−1^ NaOH solution was added until pH 12 was achieved. After stirring for 1 h, the precipitate was separated, washed with distilled water several times, and dried at 100 °C for 24 h. The dried precipitate was finely grounded before subjecting it to calcination for 24 h at 700 °C. The same protocol was adopted for the synthesis of NiFe_2_O_4_, CuFe_2_O_4_, and ZnFe_2_O_4_ using 4.75 g of NiCl_2_•6H_2_O, 4.99 g of CuSO_4_•5H_2_O, and 5.95 g of Zn(NO_3_)_2_•6H_2_O, respectively along with 16.16 g of Fe(NO_3_)_3_•9H_2_O. As-synthesized ferrites, CoFe_2_O_4_, NiFe_2_O_4_, CuFe_2_O_4_, and ZnFe_2_O_4_, were abbreviated as CoF, NiF, CuF, and ZnF, respectively.

The synthesized ferrites were characterized by various microscopic and spectroscopic techniques whose details are available in Supplementary Section 3. The photocatalytic performance of synthesized ferrites nanoparticles was evaluated by degrading MB dye in an acryl reactor with four UV lamps (8 W, *I*_max_ ~254 nm, Philips, The Netherlands) installed in a rectangular assembly. All the experiments were performed at 20 ± 2 °C (temperature was maintained by cooling fans attached at the bottom of the reactor) and at a near-neutral pH condition. For the photodegradation study, 100 mg of a ferrite photocatalyst along with 200 mL of MB solution (10 mg L^−1^) was taken in a 250 mL pyrex glass tube and subjected to UV-irradiation. After the desired contact time, 5 mL of the aqueous phase was taken out with a disposable syringe filter (Hyundai micro, Model: SN25P045NS) having a microfiltration membrane (pore size: 0.45 µm) to lock the photocatalyst. The locked photocatalyst was pumped back into the aqueous solution by pumping 2 mL of the aqueous phase through the syringe. The remaining 3 mL of the sample solution was analyzed by UV-Vis spectroscopy (LAMBDA 365 UV/Vis Spectrophotometer, Perkin Elmer) after suitable dilution. For photocatalytic degradation process, the same protocol as stated above was followed where 0.1 mL of 28% H_2_O_2_ solution was added and samples were collected at 15 s, 30 s, 45 s, 60 s, 75 s, and 300 s. Similar protocol was adopted for parameter optimization study of photocatalyst loading, dye concentration, H_2_O_2_ concentration, and UV power as well as for different dye degradation study. For the detection of active species in the photocatalytic process, 2 mL of scavenger solution (tert-butanol, EDTA, K_2_Cr_2_O_7_, *p*-benzoquinone, or KI) was added into the aqueous phase. The effect of anion on the MB degradation was studied by taking 100 mg L^−1^ of NO_3_^−^, SO_4_^2−^, Cl^−^, or CO_3_^2−^ anion in the above experiment process. For reusability study, the catalytic efficiency was measured for every cycle using the same protocol where the spent photocatalyst was separated, washed thrice with distilled water, dried in an oven at 100 °C, and used for the next catalytic cycle. The dye degradation efficiency was calculated using Eq. 14.

14 $$ \% \,dye\,degradation=\left(\frac{{C}_{0}-{C}_{t}}{{C}_{0}}\right)\times 100$$

where *C*_0_ and *C*_t_ are the initial dye concentration and dye concentration at a time ‘*t*’, respectively. All the experiments were carried out using three replicate measurements, and the standard deviation associated with data was found to be less than 5%. The data presented in figures are the average of three values, and the bar represents the standard error of the mean value.

## Supplementary Information

Supplementary Information.
