# Supplementary material for: Photocatalytic Degradation of Organic Pollutants over MFe2O4 (M = Co, Ni, Cu, Zn) Nanoparticles at Neutral pH
Source: Sci Rep. 2020 Mar 18;10:4942. doi: 10.1038/s41598-020-61930-2 (PMC7080783; doi:10.1038/s41598-020-61930-2)
Supplement: Supplementary file 1 — Supplementary Information. [file 41598_2020_61930_MOESM1_ESM.docx]

**Photocatalytic Degradation of Organic Pollutants over MFe_2_O_4_ (M = Co, Ni, Cu, Zn) Nanoparticles at Neutral pH**

Nishesh Kumar Gupta^a,b^, Yasaman Ghaffari^a,b^, Suho Kim^a,b^, Jiyeol Bae^b^, Kwang Soo Kim*^a,b^, Md Saifuddin^a,b^

*a. University of Science and Technology (UST), Daejeon, Republic of Korea*

*b. Department of Land, Water, and Environment Research, Korea Institute of Civil Engineering and Building Technology (KICT), Goyang, Republic of Korea*

**Supplementary Section 1: BET surface area analysis**

The BET surface area was calculated by the following equation:

$$\frac{\boldsymbol{1}}{\boldsymbol{W}\left[ \left( \frac{\boldsymbol{P}_{\boldsymbol{0}}}{\boldsymbol{P}} \right)\boldsymbol{-1} \right]}\boldsymbol{=}\frac{\boldsymbol{C-1}}{\boldsymbol{Q}_{\boldsymbol{m}}\boldsymbol{C}}\left( \frac{\boldsymbol{P}}{\boldsymbol{P}_{\boldsymbol{0}}} \right)\boldsymbol{+}\frac{\boldsymbol{1}}{\boldsymbol{Q}_{\boldsymbol{m}}\boldsymbol{C}}\boldsymbol{(1)}$$

where *P* is the equilibrium pressure, *P*_0_ is the saturation pressure, *Q* is the quantity of the gas adsorbed on the adsorbate, *Q*_m_ is the monolayer adsorbed gas quantity, and *C* is the BET constant. The linear BET plots (1/[*W*(*P*_0_/*P*)-1] *vs*. *P*/*P*_0_) of ferrites have been shown in **supplementary figure 5**. The unique value of *Q*_m_ was estimated from the slope and intercept of BET plots according to the following equation:

$$\boldsymbol{Q}_{\boldsymbol{m}}\boldsymbol{=}\frac{\boldsymbol{1}}{\boldsymbol{slope+intercept}}\boldsymbol{(2)}$$

The total surface area (*S*_tot_) and specific surface area (*S*_BET_) were obtained using the following equations:

$$\boldsymbol{S}_{\boldsymbol{tot}}\boldsymbol{=}\frac{\boldsymbol{Q}_{\boldsymbol{m}}\boldsymbol{Ns}}{\boldsymbol{V}}\boldsymbol{(3)}$$

$$\boldsymbol{S}_{\boldsymbol{BET}}\boldsymbol{=}\frac{\boldsymbol{S}_{\boldsymbol{tot}}}{\boldsymbol{M}}\boldsymbol{(4)}$$

where *N* is Avogadro's number, *s* (0.162 nm^2^) is the molecular cross-sectional area, *V* is the molar volume of the adsorbate gas, and *M* is the mass of the adsorbent sample.

**Supplementary Section 2: Chemicals**

Ferric nitrate nonahydrate (Fe(NO_3_)_3_•9H_2_O), Cobalt(II) chloride hexahydrate (CoCl_2_•6H_2_O), Nickel(II) chloride hexahydrate (NiCl_2_•6H_2_O), Copper(II) sulphate pentahydrate (CuSO_4_•5H_2_O), Zinc(II) nitrate hexahydrate (Zn(NO_3_)_2_•6H_2_O), Potassium iodide (KI), Sodium nitrate (NaNO_3_), Sodium sulphate (Na_2_SO_4_), Sodium chloride (NaCl), Sodium carbonate (Na_2_CO_3_), Cetyltrimethylammonium bromide (CTAB), and tert-Butanol were purchased from Samchun Pure Chemicals, Korea. Sodium hydroxide (1 N NaOH), Hydrogen peroxide (28% H_2_O_2_) solution, and Potassium dichromate (K_2_Cr_2_O_7_) were procured from Daejung Chemicals and Metals Co. Ltd, Korea. Ethylenediamine tetraacetate disodium salt (EDTA), p-Benzoquinone, TA, MB, Methyl red (MR), Methyl orange (MO), and Bromo green (BG) were supplied by Sigma Aldrich, Germany. All the chemicals were of analytical grade and used without further purification.

**Supplementary Section 3: Characterization**

The surface morphology of ferrite photocatalysts was analyzed by scanning electron microscopy (SEM) (Hitachi S-4800, Japan). The finely grounded dried samples were coated with a gold-platinum alloy by ion-sputtering (E-1048 Hitachi ion sputter). The 2D elemental mapping was performed using energy-dispersive X-ray spectroscopy (EDS) (X-Maxn 80 T, Oxford, UK). The transmission electron microscope (TEM) images were recorded with a field emission TEM (JEM-2100F, JEOL, Japan). For Brunauer-Emmett-Teller (BET) analysis, N_2_ adsorption-desorption isotherm was measured at 77 K in a Gemini series Micromeritics 2360 instrument. Before subjecting for BET analysis, samples were degassed at 473 K for 2 h with a Micromeritics FlowPrep 060. The diffraction patterns were obtained using Rigaku D/Max-2500 X-ray diffractometer (XRD) with Cu K*α* and a Ni filter where the scanning speed was set to 3º min^–1^. Fourier-transform infrared (FTIR) spectra of samples were recorded using KBr pellets over a PerkinElmer FTIR spectrometer. The recording was done with a single-beam spectrometer with 60 added scans at a 0.02 m^–1^ resolution. Ultraviolet-visible light-diffuse reflectance spectroscopy (UV-Vis DRS) spectra of ferrite samples were obtained using a Scinco-4100 spectrometer equipped with a photodiode array detector and a diffuse reflectance attachment. Before analysis, all the samples were dehydrated in the air at 673 K for 7 h. For X-ray photoelectron spectroscopic (XPS) analysis of samples, a K-alpha XPS instrument (Thermo Scientific Inc., UK) with a monochromatic Al K*α* X-ray source was used where the pressure was fixed to 4.8 x 10^–9^ mbar. The electron spin resonance (ESR) measurement was carried out by a JOEL JES-FE1C X-band spectrometer. Fluorescence spectra of TAOH samples were collected with JASCO FP-8000 Series Fluorometer with 315 nm as the excitation wavelength. Total organic carbon (TOC) was determined by the Shimadzu TOC-V CPH analyzer.


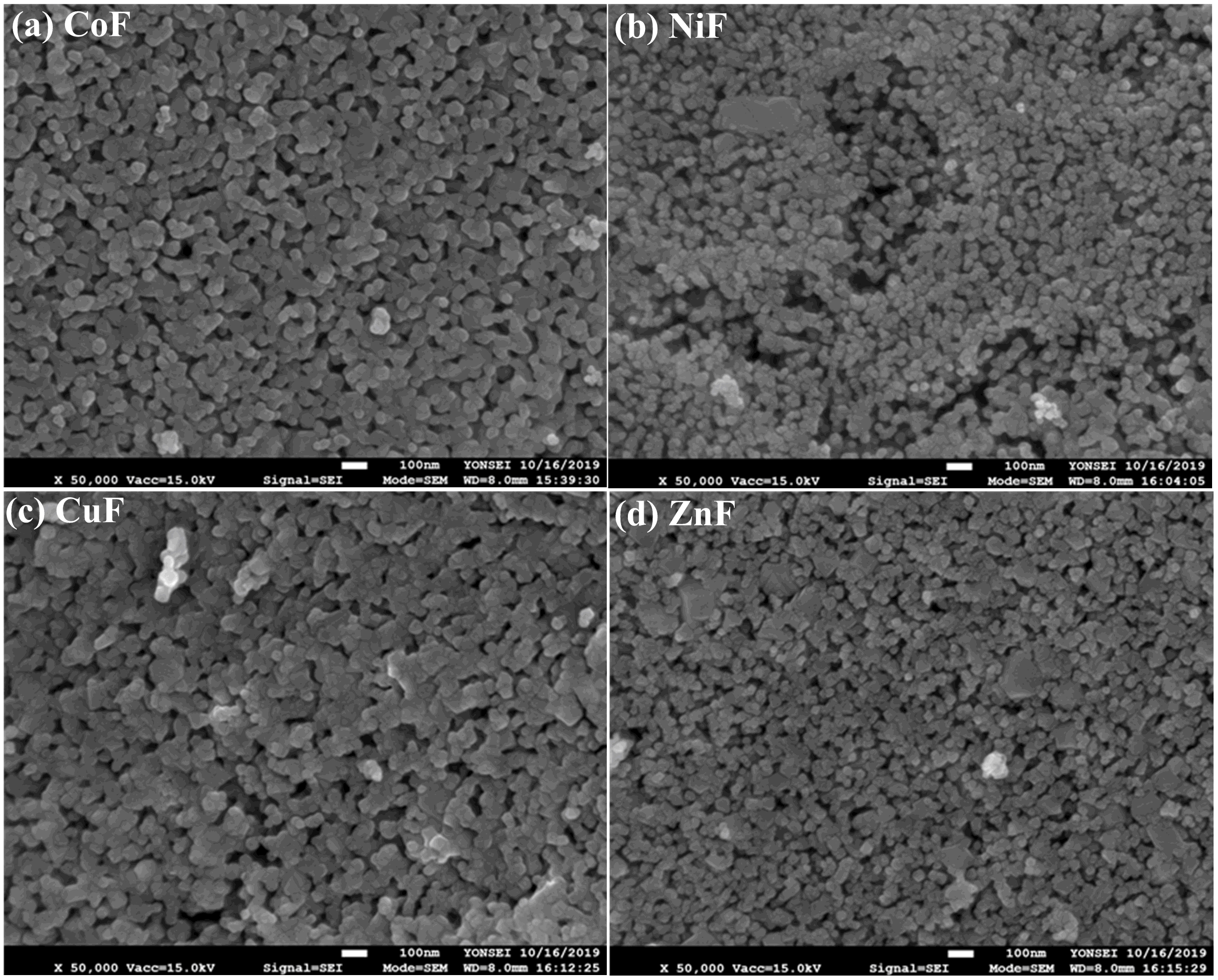


**Supplementary Figure 1.** SEM images of CoF, NiF, CuF, and ZnF photocatalysts.


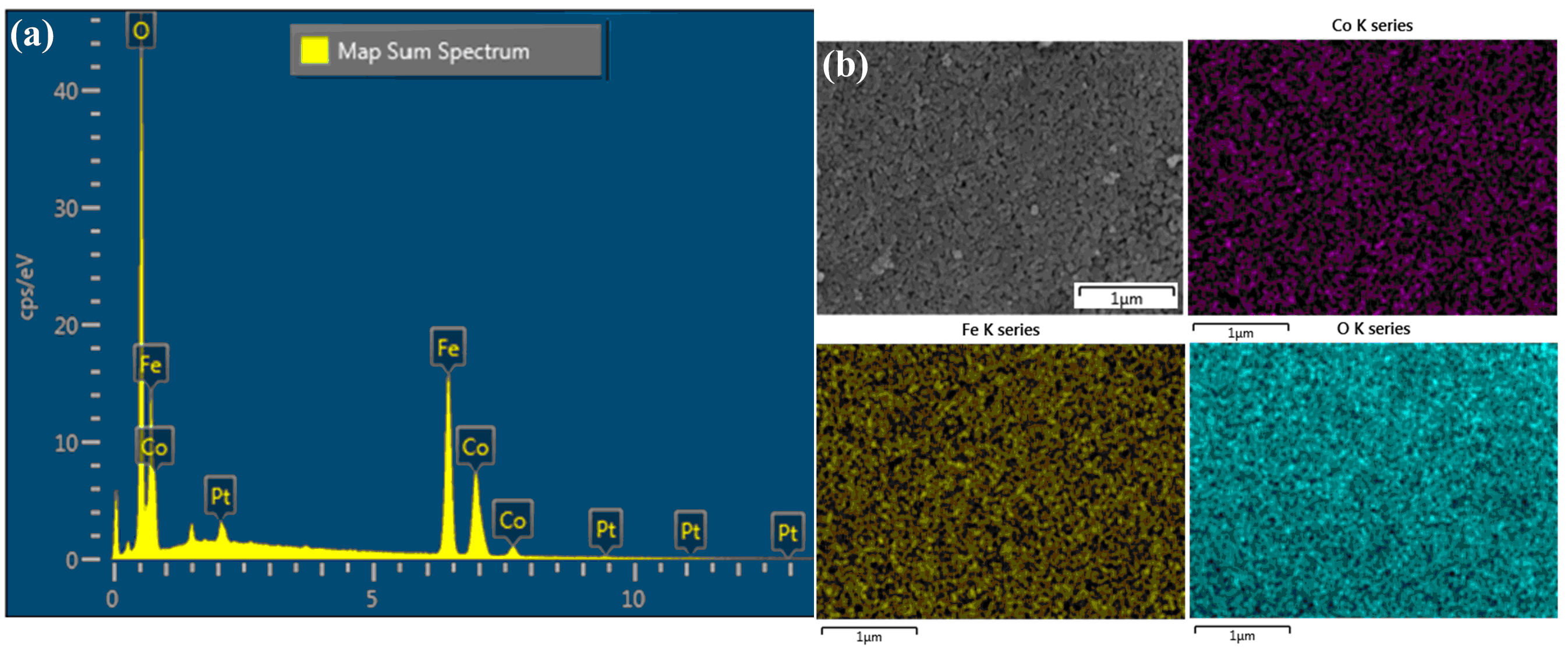


**Supplementary Figure 2.** (a) EDS spectrum and (b) 2D elemental mapping of CoF photocatalyst.

**Supplementary Figure 3.** TEM images of ferrite photocatalysts at 50 nm resolution.


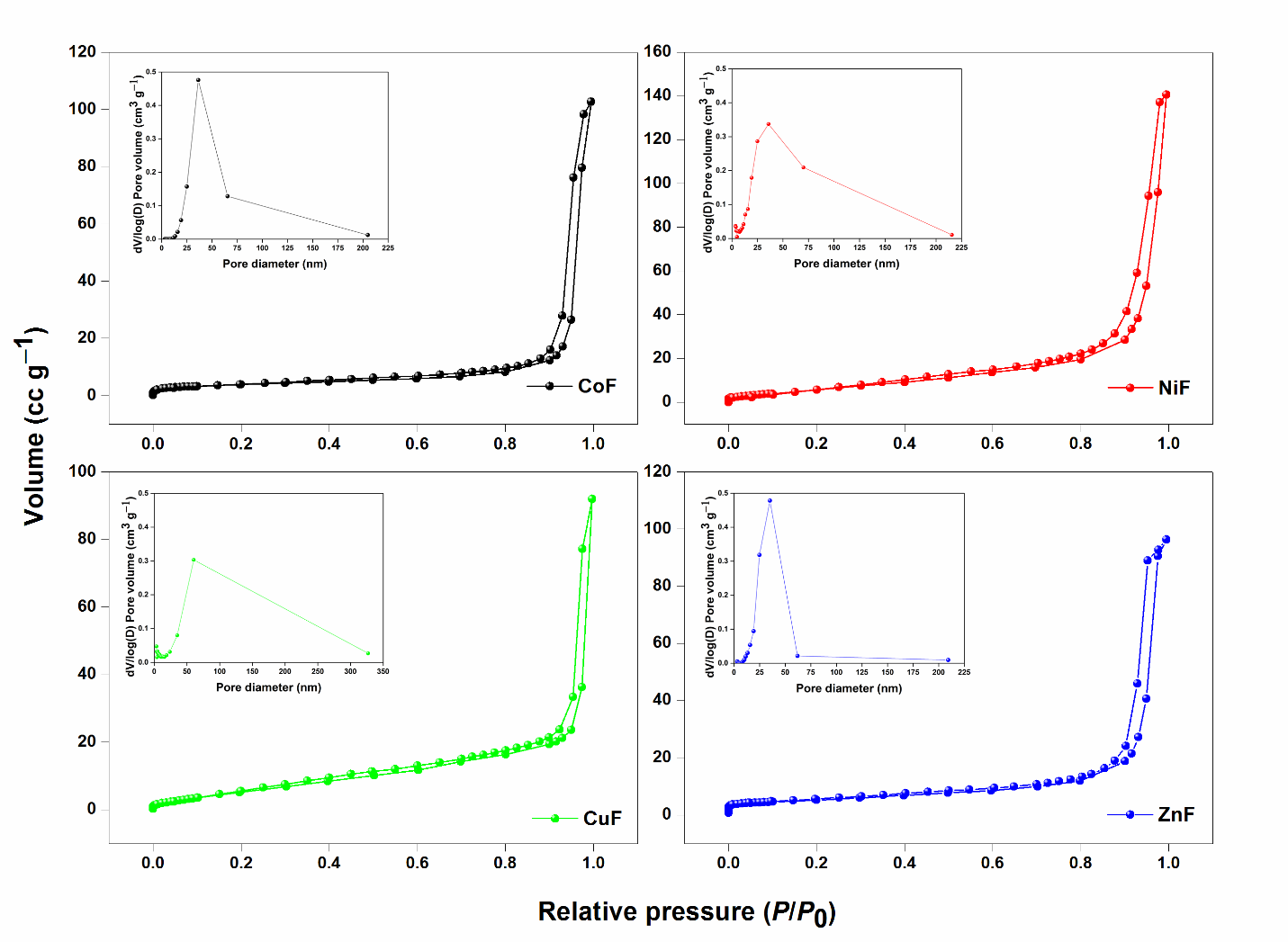


**Supplementary Figure 4.** N_2_ adsorption-desorption curve and pore size distribution curve (in inset) of CoF, NiF, CuF, and ZnF nanoparticles.


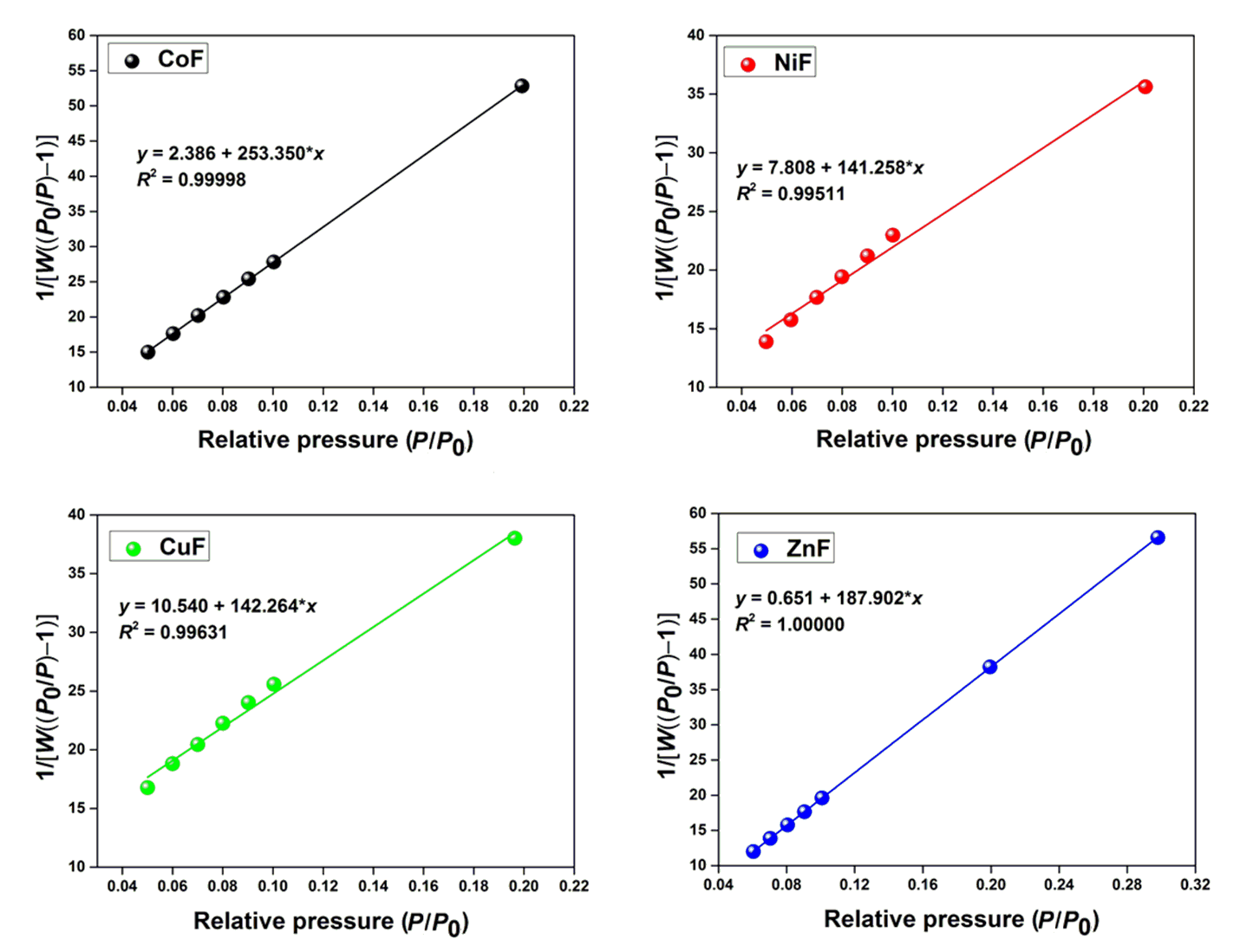


**Supplementary Figure 5.** BET plots of the ferrites.


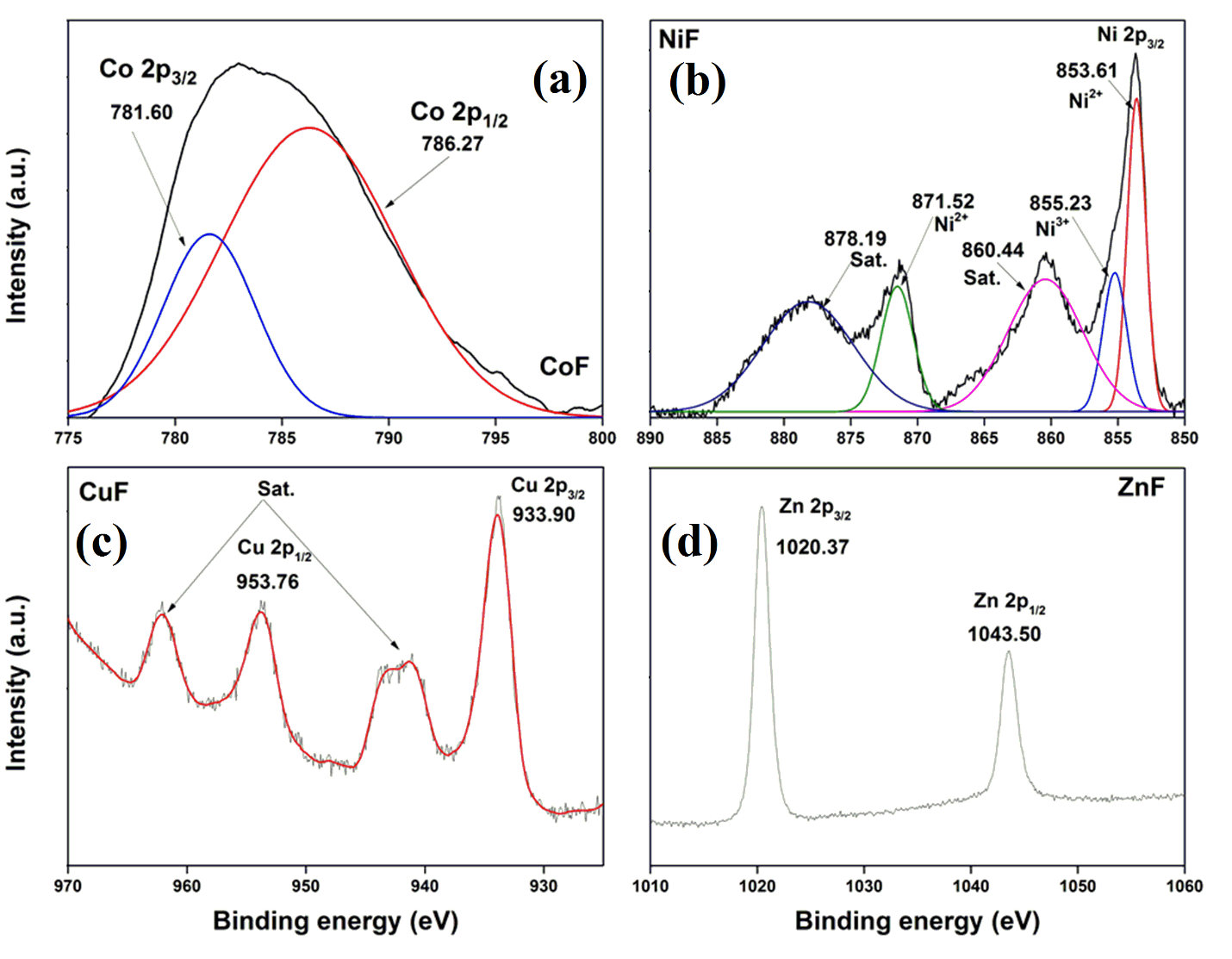


**Supplementary figure 6.** (a) HRXPS Co 2p spectrum (CoF); (b) HRXPS Ni 2p spectrum (NiF); (c) HRXPS Cu 2p spectrum (CuF); (d) HRXPS Zn 2p spectrum (ZnF).


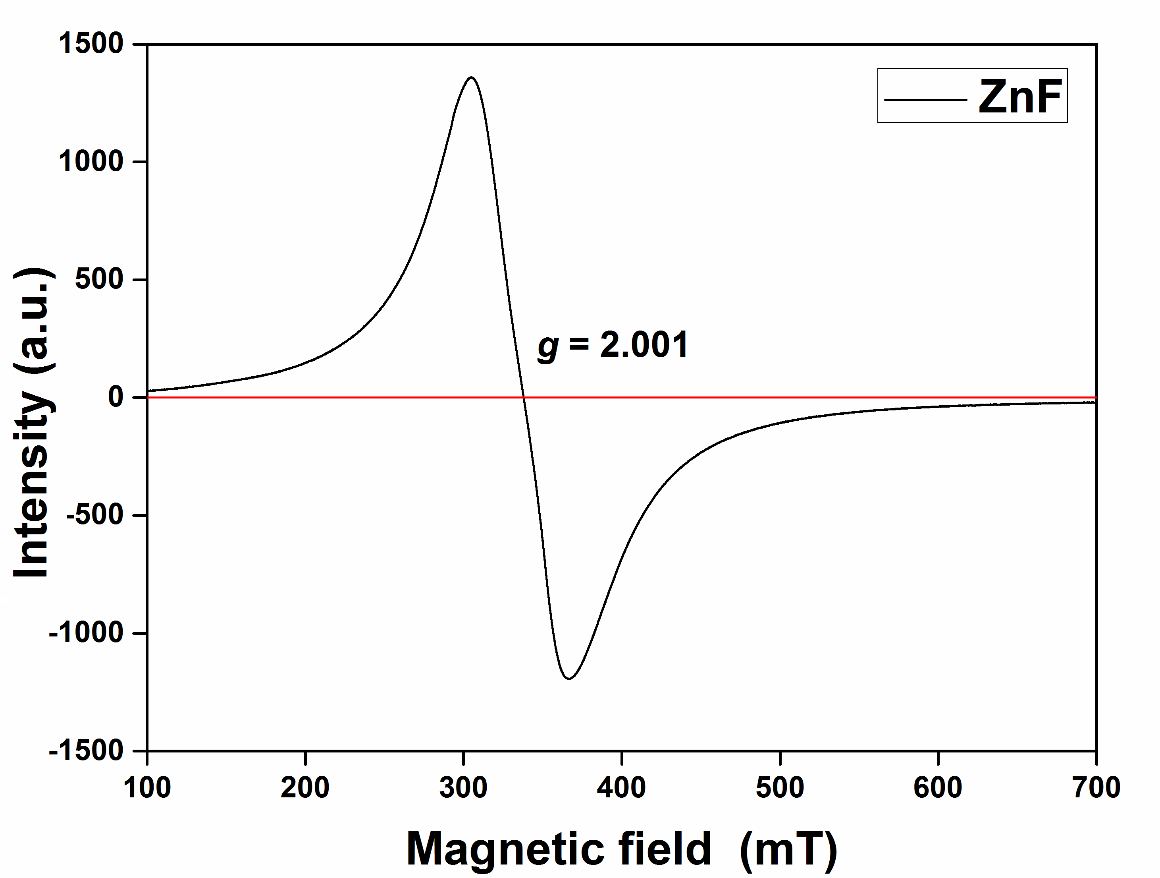


**Supplementary figure 7.** ESR spectrum of ZnF at room temperature.

**Supplementary Table 1.** Surface and porosity characteristics of ferrite photocatalysts.

| **Ferrite** | ***S*_tot_ (m^2^)** | ***S*_BET_ (m^2^ g**^–1^**)** | ***V*_p_ (cm^3^ g**^–1^**)** | ***D*_p_ (nm)** |
| --- | --- | --- | --- | --- |
| **CoF** | 5.72 | 13.62 | 0.16 | 36.38 |
| **NiF** | 9.34 | 23.36 | 0.22 | 24.91 |
| **CuF** | 6.47 | 22.79 | 0.14 | 3.41 |
| **ZnF** | 6.19 | 18.47 | 0.15 | 35.11 |

**Supplementary Table 2.** Lattice parameters and crystallite size for ferrite photocatalysts.

| **Photocatalyst** | **Lattice parameter (Å)** | ***d*_hkl_ (Å)** | **Crystallite size (nm)** |
| --- | --- | --- | --- |
| **CoF** | *a* = 8.38 | *d*_311_ = 2.53 | 34.05 |
| **NiF** | *a* = 8.34 | *d*_311_ = 2.52 | 27.33 |
| **CuF** | *a* = 5.83, *c* = 8.61 | *d*_211_ = 2.50 | 15.84 |
| **ZnF** | *a* = 8.45 | *d*_311_ = 2.55 | 35.99 |

**Supplementary Table 3**. Kinetic parameters for MB degradation over different ferrite photocatalysts

| **Photocatalyst** | ***k*_app_ (min**^–1^**)** | ***t*_1/2_ (s)** | ***R*^2^** |
| --- | --- | --- | --- |
| **CoF** | 2.141 ± 0.006 | 19.417 | 0.999 |
| **NiF** | 2.417 ± 0.003 | 17.200 | 0.999 |
| **CuF** | 2.065 ± 0.002 | 20.134 | 1.000 |
| **ZnF** | 2.069 ± 0.003 | 20.099 | 0.999 |
